# Zigzag Hollow Cracks of Silver Nanoparticle Film Regulated by Its Drying Micro-environment

**DOI:** 10.1186/s11671-018-2777-x

**Published:** 2018-11-06

**Authors:** Ruiqiang Tao, Jianhua Zhang, Zhiqiang Fang, Honglong Ning, Jianqiu Chen, Caigui Yang, Yicong Zhou, Rihui Yao, Yongsheng Song, Junbiao Peng

**Affiliations:** 1 State Key Laboratory of Luminescent Materials and Devices, South China University of Technology, Guangzhou, 510640 China; 2 Key Laboratory of Advanced Display and System Applications, Ministry of Education, Shanghai University, Shanghai, 200072 China; 3 State Key Laboratory of Pulp and Paper Engineering, South China University of Technology, Guangzhou, 510640 China; 4 Guangdong Fenghua Advanced Technology Holding Co., LTD, Zhaoqing, 526020 China

**Keywords:** Particles, nanosize, Sol-gel preparation, Solidification, Surfaces, Thin films

## Abstract

We first verify the critical impact of evaporation on the formation of zigzag hollow cracks by regulating the drying micro-environment of silver nanoparticle film. Uneven evaporation and component segregation contributes to the flows along the surface and inside of droplets. Asymmetric vapor concentration distribution is capable of weakening the surface flow of droplets, thus suppressing the inner compressive stress of nanoparticles and leading to a surface morphology with less cracks. Although defect-free and surface smooth nanoparticle film deposited by a solution-based method remains a big challenge, our work has referential significance to optimize high-quality nanoparticle film with appropriate deposition and curing processes. Moreover, an optimization possibility through the drying micro-environment should be considered in high-end applications due to its enhanced effect on high-resolution patterns.

**Electronic supplementary material:**

The online version of this article (10.1186/s11671-018-2777-x) contains supplementary material, which is available to authorized users.

## Introduction

Precursor inks (silver, gold, copper, etc.) are compatible with flexible fabrication due to their low processing temperature (< 200 °C) [1]. However, crack problem remains unsolved and will deteriorate the conductivity and adhesion properties of the deposited film [2]. The underlying mechanism is worth further exploring, while most previous reports concentrate on some external effect, such as laser [3], intense pulsed light [4], and ions [5]. Uneven evaporation nature is underrated to some extent, although the coffee ring effect has been proven in numerous studies [6]. Fast evaporation flux of the periphery area and the pinning of the triple line contribute to the outward compensation flow inside of droplets. Accordingly, directional surface flow can be induced with component segregation [7].

Evaporation dynamics, chemical reduction, microfluidic regulation, and nanoparticle assembly have been discussed here to achieve a comprehensive understanding of the crack-forming process. To explore the critical impact of the drying micro-environment on the forming of zigzag hollow cracks, the coffee ring effect is enhanced by the ink formulation, so as to (1) drive nanoparticles to the periphery area and make them self-assembled to form the surface film, (2) promote the forming of cracks by increasing the compressive stress, (3) increase the air pressure between two neighboring droplets, which avoids their coalescence and leads to a self-aligning phenomenon, making the distance of the droplet boundaries short enough to present the obvious effect of the drying micro-environment.

The regulation of the drying micro-environment directly proves the close relationship between the forming of cracks and solvent evaporation. It has certain innovations and advantages in determining the critical impact of evaporation on the forming of surface cracks, while other factors are controlled to be unchanged. According to the proposed mechanism, wet film cured without the forming of cracks has been achieved here by enhancing the chemical reduction, or by reducing the size of droplets using inkjet printing technology. This work has referential significance to optimize high-quality nanoparticle film deposited using solution-based methods.

## Materials and Methods

**Fig. 1:**
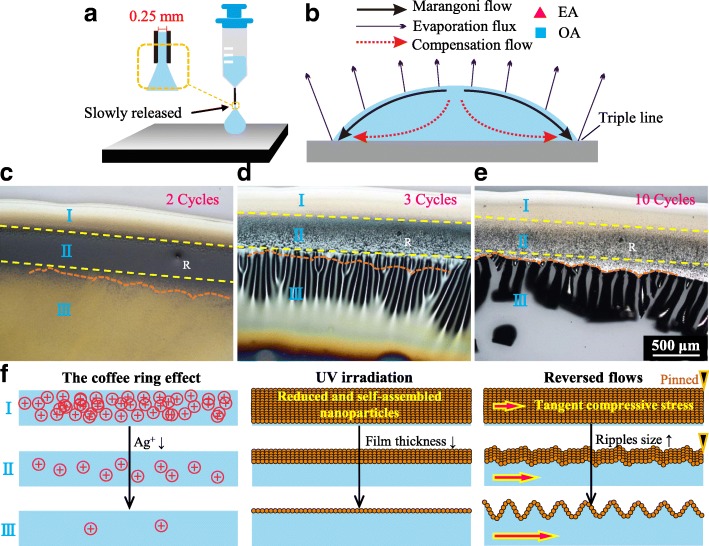
Crack formation process. **a** Droplet released by a syringe. **b** Schematic of the induced directional flows. **c**, **d**, **e** The released droplets followed by cycles of UV irradiation. **f** Schematic of different morphologies for different regions

Silver acetate (2.5 g), ethyl alcohol (EA, 3 ml), and Octylamine (OA, 3 ml) are mixed with stirring at room temperature for 2 h. The prepared ink is filtered (0.22 μm) before using. Glass substrate is cleaned by DI water, isopropyl, and tetrahydrofuran in an ultrasonic cleaner for 10 min in sequence. A syringe with a nozzle diameter of 0.25 mm is used to release droplets (*d* ~ 5 mm) (Fig. 1a). The increased drying time of large size droplets (*t*_drying_ ~ *r*^2^) makes the observation easier. Hotplate and UV equipment (IntelliRay 600 W, Uvitron, USA) are used to promote chemical reduction with different evaporation dynamics. The UV equipment is equipped with a light filter, which eliminates its hydrophilic effect. Surface morphology was observed with an optical microscope up to 1000× (Nikon Eclipse E600 POL) and a scanning electron microscope (SEM, NOVA NANOSEM 430) installed with an energy-dispersive X-ray spectrometer (EDS) module.

## Results and Discussion

The coffee ring effect and the induced Marangoni flow are schematically described in Fig. 1b. The OA/EA ratio increases at the periphery area, on account of the higher evaporation rate, as well as the higher surface tension and boiling point of OA (28 dyn/cm, 176 °C) than EA (22 dyn/cm, 78 °C). The surface tension difference results in an outward Marangoni flow. Three different regions (I, II, and III) appear after 2 cycles of UV irradiation (60 s/cycle) (Fig. 1c). The intervals of each cycle are used to remove the thermal effect. Solutes aggregate at region I due to the outward compensation flow and is solidified soon because of the fierce evaporation. Regions II and III are nanoparticle suspensions, but the latter is more sparse. More cycles of irradiation make region III transformed from ripples (3 cycles) to cracks (10 cycles), while region II is rough, and region I keeps smooth (Fig. 1d, e). The adhesion property is seriously deteriorated when cracks are formed. Figure 1f schematically describes the underlying mechanism. Monodispersed nanoparticles (Additional file 1: Figure S1) tend to be self-assembled and form compact surface film due to the outward Marangoni flow, the evaporation driving up force, and the surface tension (large specific surface area). The film thickness decreases from region I to III, accordingly making the strains increased under compressive stress, and even radial ripples can be resulted. The periphery surface film suppresses the evaporation of the underneath liquid, thus the compensation flow is reversed, leading to the drop of the liquid level, and inducing a compressive stress in chord direction.

**Fig. 2:**
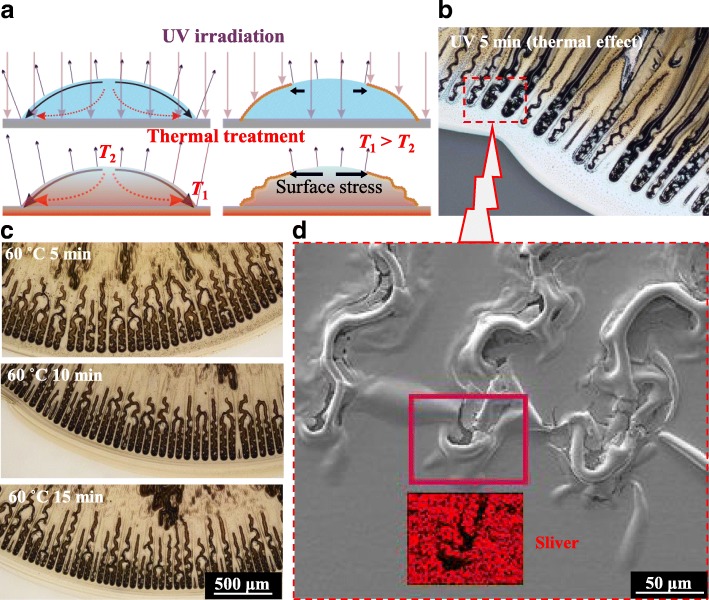
Zigzag hollow cracks. **a** Schematic of the difference between UV irradiation and thermal treatment for the forming of surface nanoparticle film. **b** Zigzag-shaped ripples obtained with UV irradiation for 5 min. **c** More regular ripples obtained at a heated glass substrate at 60 °C for 5 to 15 min. **d** SEM-EDS measurements

Solution-processed films cured by UV irradiation have the weaker coffee ring effect due to its moderate evaporation rate than thermally treated ones [8]. It contributes to the difference on the formation of surface films (Fig. 2a). Thermal effect should be considered when wet film is continuously UV irradiated for 5 min, resulting in zigzag-shaped ripples at the periphery area (Fig. 2b). The deformation in chord direction originates from the increased radial compressive stress, which is induced by the enhancing of the outward surface flow and the evaporation difference. More regular zigzag-shaped ripples can be observed when a moderate temperature is applied to the substrate (*T*_*s*_ = 60 °C). The sintering time (5 to 15 min) independence of ripples demonstrates their forming before being completely solidified (Fig. 2c). Liquid-supported surface thin film is easily deformable under the compressive stress, and cracks generate along the ripples (Fig. 2d). As the drying process continues, the reversed compensation flow will leave a hollow inside topography of ripples, which can be evidenced by the EDS area scanning for silver element.

**Fig. 3:**
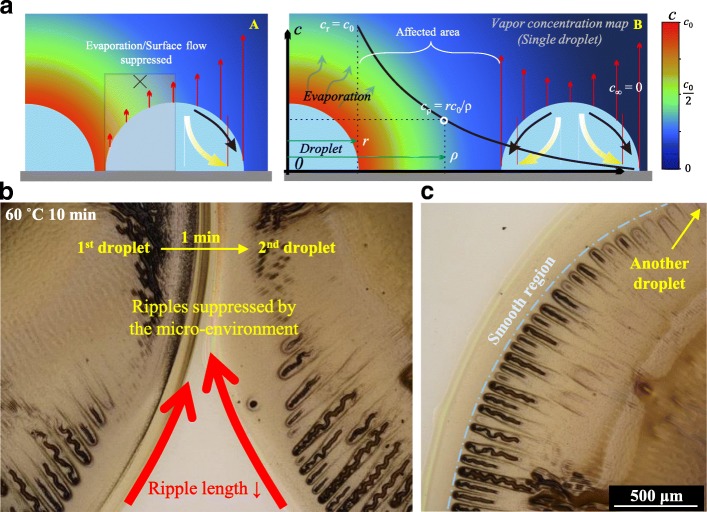
Zigzag hollow cracks regulated by its drying micro-environment. **a** Color map of the drying micro-environment based on the simplest vapor diffusion model. **b** Effect of the drying micro-environment on two subsequently released droplets with a short distance. **c** Ripples change from the nearest region to the farther region of two neighboring droplets

The critical impact of evaporation on the forming of cracks has been discussed above. The drying micro-environment is capable of regulating the distribution of evaporation flux, which is studied in-depth in our previous report [9, 10], and therefore is also likely to have an impact on crack formation. Based on the simplified vapor diffusion model of solvent evaporation (*c*_*ρ*_ = *rc*_0_/*ρ*), a color map of the vapor concentration (*c*) can be drawn to describe the influences of the drying micro-environment on the evaporation of two neighboring droplets (Fig. 3a). Asymmetrical evaporation flux can be achieved when another droplet is released nearby. A closer distance of droplet boundaries suppresses the evaporation and the surface flow [11] (Additional file 1: Figure S2), accordingly reducing the tendency to form ripples, especially zigzag-shaped ones. Outward surface flow increases the air pressure between droplets, thus making them self-aligned to achieve a short distance of only tens of microns. Even no ripples formed at the nearest region, and then the ripple length increases and finally recovers to zigzag shape with the increased distance of droplet boundaries (Fig. 3b, c). The area of the smooth periphery region enlarges due to the more time for nanoparticle reduction and aggregation before they are self-assembled to form thick film under the premise of evaporation suppression. Furthermore, the suppression effect is more apparent for the first droplet, which is released 60 s earlier than the second one. The earlier formed surface film of the first droplet diminishes its evaporation effect on the drying micro-environment of the second droplet, while the evaporation of the second droplets will influence the whole ripple-forming process of the first droplet.

It should be emphasized that regulation of the drying micro-environment not only acts as a method to suppress zigzag hollow cracks but also directly proves the close relationship between the forming of cracks and solvent evaporation. This work has referential significance to optimize high-quality nanoparticle film, especially for precursor ink. When droplets are still released by the syringe, the cracks can be easily removed by enhancing the rate of chemical reduction under the premise that the evaporation is less affected (Additional file 1: Figure S3). A thin surface film on liquid, which can be easily deformed, can form under the action of evaporation, when the reduced nanoparticles are few. Therefore, the accelerated chemical reduction will make the solute concentration high enough to form a thick self-assembled surface nanoparticle film and then avoid the forming of cracks. Another effective way to deal with the cracks can be achieved by reducing the size of droplets (Additional file 1: Figure S4). Inkjet printing is a potential technique to deposit wet film consisting of tiny droplets (diameter ~ 50 μm). Inkjet-printed films using the same ink system can be solidified without ripples and cracks, even cured at a high temperature of 100 °C for 30 min, taking advantages of [1] the quicker solidification process, [2] the weaker local evaporation rate, [3] the weaker fluid flows, [4] the higher local solute concentration, and [5] the changed drying micro-environment of each droplet.

## Conclusion

The critical impact of evaporation on the forming cracks of solution processed nanoparticle film has been studied considering various aspects. The thickness of the liquid-supported surface film formed during the solidification process has a major influence on the topography under compressive stress. The size and shape of ripples can be continuously regulated by changing its drying micro-environment. This work provides a feasible way to accurately suppress the surface cracks and may have referential significance to optimize high-quality nanoparticle film deposited using solution-based methods.

## Additional file

Additional file 1: **Figure S1.** Monodispersed nanoparticles obtained after UV irradiation for 60 s. **Figure S2.** Schematic of asymmetrical directional flows of two neighboring droplets. **Figure S3.** High-quality nanoparticle film optimized with enhanced chemical reduction. (a) Schematic of wet film cured with different distance (8 cm, 24 cm) from the UV lamp. The side wall of the curing box is specially designed to achieve uniform UV irradiation, whose strength can be significantly increased when a closer distance from the UV lamp is applied (E_1_<<E_2_). (b) Syringe released droplets cured with cycles of UV irradiation at a distance of 24 cm from the UV lamp. (c) Syringe released droplets cured with cycles of UV irradiation at a distance of 8 cm from the UV lamp. **Figure S4.** High-quality nanoparticle film optimized using an inkjet printer (DMP-2831, FUJIFILM Dimatix, USA) with nozzle diameter of 16 μm. (a) Microscope photo with a magnification of 100×, and (b) 3D profile of the deposited film thermally cured at 100 ˚C for 30 min. The surface fluctuation can be ascribed to the travel line of inkjet printing with set drop space of 35 μm. (DOCX 1720 kb)

## Abbreviations

DI: Deionized
EA: Ethyl alcohol
EDS: Energy-dispersive X-ray spectrometer
OA: Octylamine
SEM: Scanning electron microscope
UV: Ultraviolet
