# Supplementary material for: Zigzag Hollow Cracks of Silver Nanoparticle Film Regulated by Its Drying Micro-environment
Source: Nanoscale Res Lett. 2018 Nov 6;13:354. doi: 10.1186/s11671-018-2777-x (PMC6219994; doi:10.1186/s11671-018-2777-x)
Supplement: Supplementary file 1 — Figure S1. Monodispersed nanoparticles obtained after UV irradiation for 60 s. Figure S2. Schematic of asymmetrical directional flows of two neighboring droplets. Figure S3. High-quality nanoparticle film optimized with enhanced chemical reduction. (a) Schematic of wet film cured with different distance (8 cm, 24 cm) from the UV lamp. The side wall of the curing box is specially designed to achieve uniform UV irradiation, whose strength can be significantly increased when a closer distance from the UV lamp is applied (E1<<E2). (b) Syringe released droplets cured with cycles of UV irradiation at a distance of 24 cm from the UV lamp. (c) Syringe released droplets cured with cycles of UV irradiation at a distance of 8 cm from the UV lamp. Figure S4. High-quality nanoparticle film optimized using an inkjet printer (DMP-2831, FUJIFILM Dimatix, USA) with nozzle diameter of 16 μm. (a) Microscope photo with a magnification of 100×, and (b) 3D profile of the deposited film thermally cured at 100 ˚C for 30 min. The surface fluctuation can be ascribed to the travel line of inkjet printing with set drop space of 35 μm. (DOCX 1720 kb) [file 11671_2018_2777_MOESM1_ESM.docx]

**Supporting Information**

Zig-zag Hollow Cracks of Silver Nanoparticles Film Regulated by its Drying Micro-environment

*Ruiqiang Tao^1^, Jianhua Zhang^2^, Zhiqiang Fang^3^, Honglong Ning^1*^, Jianqiu Chen^1^, Caigui Yang^1^, Yicong Zhou^1^, Rihui Yao^1*^, Yongsheng Song^4^, Junbiao Peng^1^*

*1. State Key Laboratory of Luminescent Materials and Devices, South China University of Technology, Guangzhou 510640, China*

*2. Key Laboratory of Advanced Display and System Applications of Ministry of Education, Shanghai University, Shanghai 200072, China*

*3. State Key Laboratory of Pulp and Paper Engineering, South China University of Technology, Guangzhou 510640, China*

*4. Guangdong Feng Hua Advanced Technology Holding CO., LTD, Zhaoqing 526020, China*

**Supporting Figures**

**Note 1**. Nanoparticles of the deposited film using the developed ink.


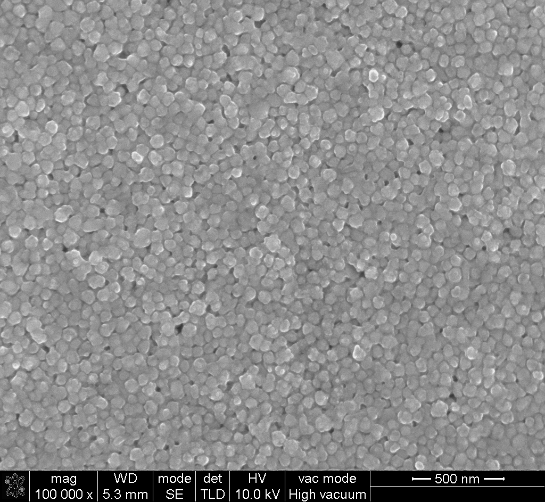


**Figure S1**. Monodispersed nanoparticles obtained after UV irradiation for 60 seconds.

**Note 2.** Evaporation regulated by the drying micro-environment


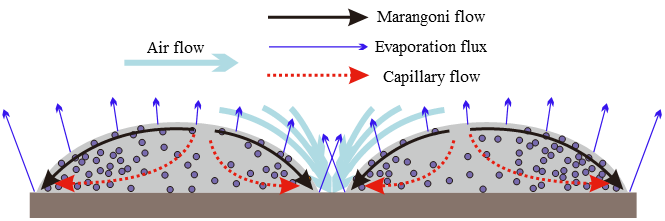


**Figure S2.** Schematic of asymmetrical directional flows of two neighboring droplets.

**Note 3.** Two simple ways provided to optimize high quality nanoparticles film.


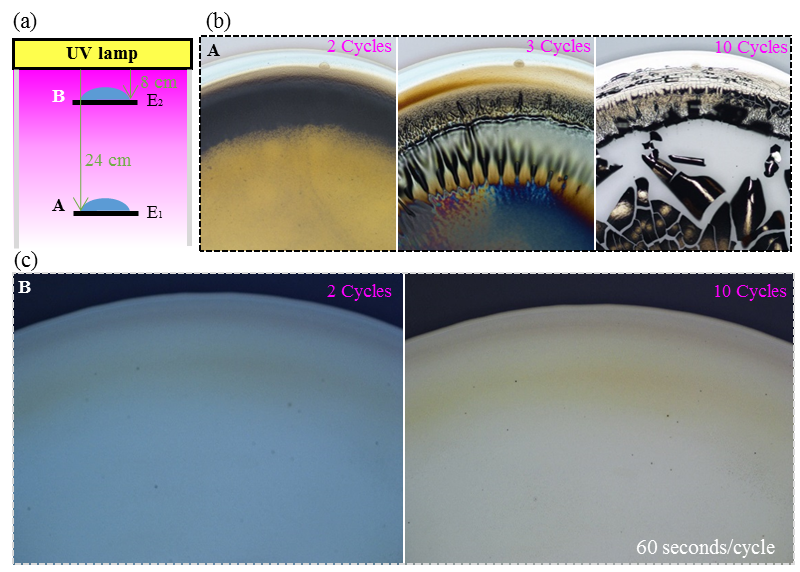


**Figure S3.** High quality nanoparticles film optimized with enhanced chemical reduction. (a) Schematic of wet film cured with different distance (8cm, 24cm) from the UV lamp. The side wall of the curing box is specially designed to achieve uniform UV irradiation, whose strength can be significantly increased when a closer distance from the UV lamp is applied (E_1_<<E_2_). (b) Syringe released droplets cured with cycles of UV irradiation at a distance of 24 cm from the UV lamp. (c) Syringe released droplets cured with cycles of UV irradiation at a distance of 8 cm from the UV lamp.

When the distance is set to 24 cm, the UV light intensity is weak, and the reduced nanoparticles are few, then a very thin surface film on liquid can form under the action of evaporation. Accordingly, ripples are easily formed, and will turn into cracks after cycles of UV irradiation. In contrast to the above phenomenon, when the wet film is closer to the UV lamp, the UV light intensity is stronger, which accelerates the chemical reduction, while the evaporation is less affected.


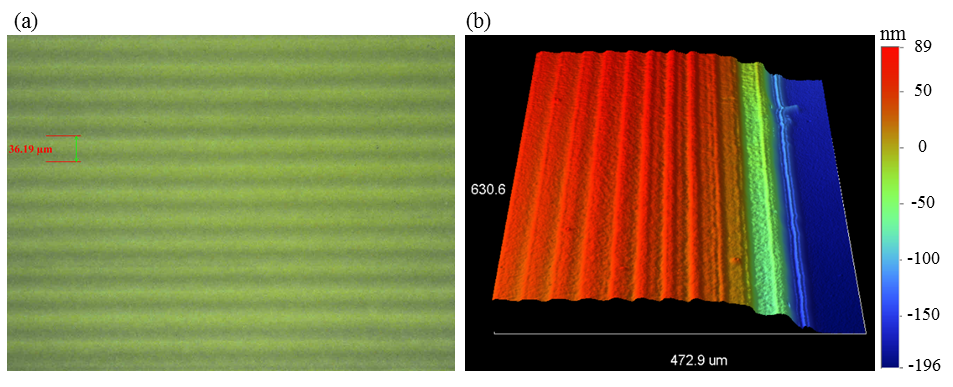


**Figure S4.** High quality nanoparticles film optimized using an inkjet printer (DMP-2831, FUJIFILM Dimatix, U.S.A.) with nozzle diameter of 16 μm. (a) Microscope photo with a magnification of 100 x, and (b) 3D profile of the deposited film thermally cured at 100 ˚C for 30 minutes. The surface fluctuation can be ascribed to the travel line of inkjet printing with set drop space of 35 μm.

A liquid film consisting of a single layer of tiny droplets has the following features to avoid ripples and cracks.

(1) The reduction of the droplet size shortens the curing time per unit volume of the droplet, and thus it is easier to be solidified before the surface nanoparticles film is formed;

(2) Although the curing time of smaller sized droplet is shortened, the local evaporation rate is weakened, thus the timing of the forming of self-assembled surface film will be delayed;

(3) Smaller sized droplet has a reduced evaporation rate gradient along its surface, which leads to weaker fluid flows. Therefore, cracks will not form even a higher curing temperature for the wet film is adopted.

(4) The thickness of the wet film is limited by the height of a single droplet, which is generally only a few micrometers. Under this premise, both the thermal and the UV curing can quickly increase the local solute concentration;

(5) When a plurality of droplets are simultaneously released to form a thin film, the drying micro-environment of each droplet is actually affected by the vapor diffusion of the surrounding droplets, thus suppressing the forming of ripples and cracks.
